# Supplementary material for: Combining rVAR2 and Anti-EpCAM to Increase the Capture Efficiency of Non-Small-Cell Lung Cancer Cell Lines in the Flow Enrichment Target Capture Halbach (FETCH) Magnetic Separation System
Source: Int J Mol Sci. 2024 Sep 11;25(18):9816. doi: 10.3390/ijms25189816 (PMC11432156; doi:10.3390/ijms25189816)
Supplement: Supplementary file 1 [file ijms-25-09816-s001.zip › ijms-3176031-supplementary.pdf]

# Supplementary

## Combining rVAR2 and Anti-EpCAM to Increase the Capture Efficiency of Non-Small Cell Lung Cancer Cell Lines in the Flow Enrichment Target Capture Halbach (FETCH) Magnetic Separation System

*Optimization of rVAR2 Concentration*

The concentration of rVAR2 plays an important role in the characterization of ofCS on tumor cells as well as in the recovery of tumor cells from the blood. To determine the optimal concentration, different concentrations of rVAR2 (ranging from 0 nM to 200 nM) were incubated with cancer cell lines (PC3 and LNCaP cells). The results showed that within the range of 0 nM to 100 nM, the fluorescence signal increased with increasing concentrations of rVAR2. Within the range of 100 nM to 200 nM, no further increase in signal was observed, indicating maximal binding (see Figure S1). Therefore, 100 nM was chosen as the optimal concentration for rVAR2, consistent with findings from previous studies on A549 cells[23].

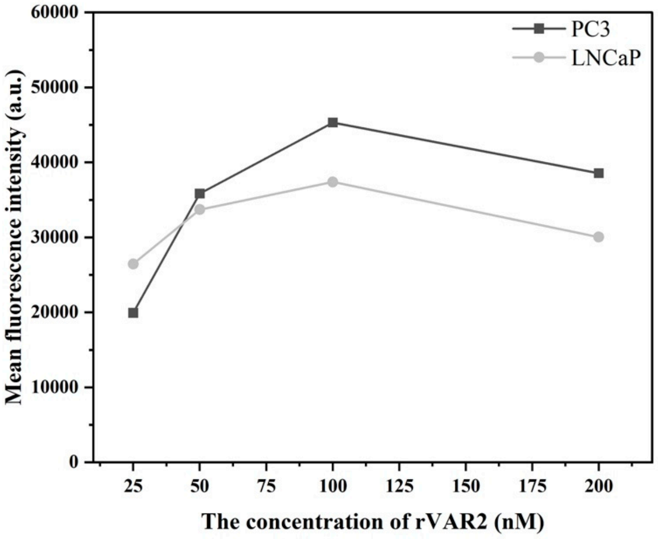


**Figure S1.** Assessment of rVAR2 binding to prostate cancer cell lines PC3 and LNCaP using flow cytometry with a PE-conjugated anti-V5 antibody. Mean fluorescence intensity was quantified after incubating cells with varying concentrations of rVAR2. The graph shows the relationship between rVAR2 concentration and fluorescence intensity, indicating the optimal binding concentration for each cell line.

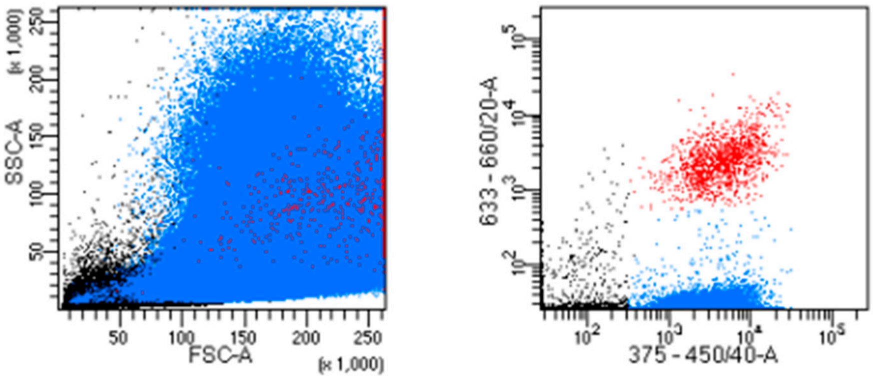


**Figure S2.** FACS plot of a captured sample tube used as a reference. The red population represents tumor cells, while the blue population indicates white blood cells.
